# On-Demand Photopolymerization of Fiber-Reinforced Polymers Exhibiting the Shape Memory Effect

**DOI:** 10.3390/polym13244300

**Published:** 2021-12-09

**Authors:** Xavier Allonas, Johann Pierrel, Ahmad Ibrahim, Céline Croutxé-Barghorn

**Affiliations:** Laboratory of Macromolecular Photochemistry and Engineering, Université de Haute Alsace, 3b Rue Alfred Werner, 68093 Mulhouse, France; johann.pierrel@uha.fr (J.P.); AIbrahim@gcttg.com (A.I.); celine.croutxe-barghorn@uha.fr (C.C.-B.)

**Keywords:** shape memory polymers, Michael addition, photopolymerization

## Abstract

Fiber-reinforced polymers exhibiting the shape memory effect were created on the basis of a one-pot three-step chemical process. The first step is a Michael addition, which creates linear polymer chains. The second step is free radical photopolymerization, which increases the degree of curing of polymers. The last step is post-consolidation due to the reaction of previously formed secondary amines on the residual double bonds. By employing such chemistry to impregnate glass fibers, the final composite exhibits a convincing shape memory effect, as shown by cyclic thermomechanical tests.

## 1. Introduction

Shape memory polymers (SMPs) have attracted an increasing level of interest over the past 20 years due to their unique properties. Although particularly easy to produce, these polymers exhibit lower mechanical strength and restitution stress than shape memory alloys (SMAs). Therefore, reinforcements have been incorporated into these resins in order to improve their mechanical properties, thus giving rise to shape memory composites (SMCs) [1,2,3,4,5]. These SMCs can then be divided into two different categories depending on the use of particles or fibers as reinforcement [6]. When using fibers, it is possible to create deployable pieces such as antennas or solar panels, which would be compacted on Earth and then unfolded in space [2]. A limited number of studies deal with SMCs reinforced with unidirectional fibers, sandwich structures or with a single layer of woven fibers [2,5,7,8]. Few publications show results obtained on SMCs manufactured using several layers of continuous fibers, although limited information is given about the fixity rate which is essential to characterize the SME [9].

The creation of SMPs via a photopolymerization process has already been proposed. [10,11,12,13,14,15,16,17] In particular, a photopolymerized system based on the addition of thiol onto double bonds has led to a high number of studies in this field [10,12,13,14,15,16]. However, the use of photopolymerization as a curing process for the fabrication of woven fiber SMCs has not been developed so far, while this on-demand process presents many advantages over traditional curing processes, such as high productivity, good reproducibility and cost efficiency.

Therefore, it could be interesting to study the shape memory effect (SME) of samples made with several layers of woven fibers. Following the development of a new material for the creation of SMPs [10,11,12,13,14,15,16,17,18,19,20], it was decided to use the combination of a Michael addition followed by radical photopolymerization to obtain polymers reinforced with several layers of woven glass fibers. This methodology would allow for the formation of SMCs in a relatively fast time scale by using photopolymerization to cure the resin. This would thus make it possible to diversify the nature of the materials manufactured and therefore to extend the use of SMCs to new areas.

## 2. Materials and Methods

Four fiber-reinforced composites were prepared by impregnating ten layers of woven glass fibers (206 g/m^2^) with different formulations (50 wt% of formulation and 50 wt% of fibers) as follows: The first composite (Run **a**) is based on the photopolymerization of bisphenol A ethoxylated diacrylate (EBAD, SR 349 from Sartomer, Paris, France) in the presence of 2.5 wt% of bis(2,4,6-trimethylbenzoyl)phenylphosphine (BAPO, IGM resins, Gerenzano, Italy) as a photoinitiator. Run **b** is based on thio–Michael/free radical photopolymerization reactions and is composed of the same resin as in Run **a,** in which 1,5-pentanedithiol (DT, Merck, Darmstadt, Germany) was added at a functional ratio of 1:4 with respect to the acrylate groups. In addition, 0.8 wt% of triethylamine (TEA, Merck, Darmstadt, Germany) was added as a catalyst for the thio–Michael addition. Run **c** and Run **d** were composed of the same resin as in Run **a,** in which 1,5- pentanediamine (DA, Merck, Darmstadt, Germany) was added at a functional ratio of 1:4 with respect to the acrylate groups.

**Scheme 1:**
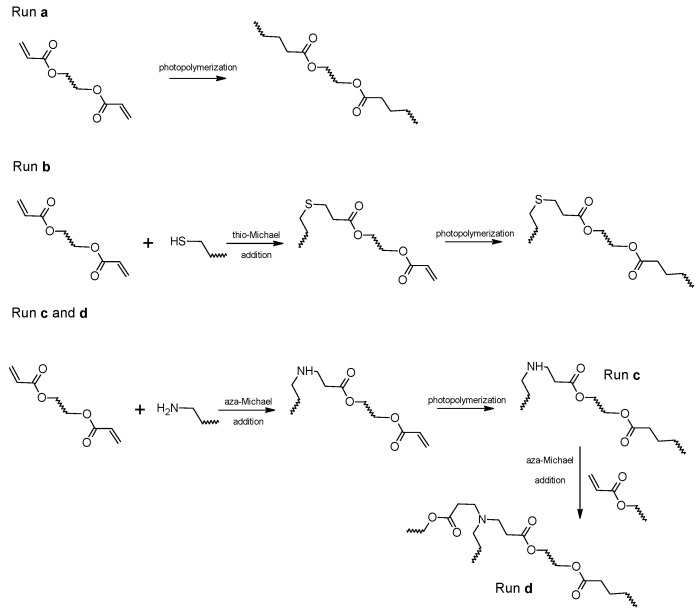
Mechanisms of reactions involved in the fabrication of the four samples.

In the case of Run **a**, irradiation was operated after a 30 min compaction step under a vacuum. Run **b** to Run **d** were first left at room temperature in order to promote the Michael addition between either the thiol or the amine and the acrylate, leading to an increase in the viscosity of the system (Step 1 in Scheme 1). After 270 min or 50 min for the systems DT-EBAD or DA-EBAD, respectively, the composite was compacted under a vacuum for 30 min. At this point, the whole thiol or amine has reacted with ¼ of the acrylate functions, forming a linear prepolymer. Then, in a second step, the system was irradiated at 395 nm for 2.5 s at 2 W/cm^2^, i.e., a total light dose of 5 J/cm^2^. During this step, most of the acrylate functions are polymerized through a free radical photopolymerization reaction initiated by the photodecomposition of BAPO (Step 2 in Scheme 1). In the case of Run **c** and Run **d**, the sample was left in the dark for 1 day (Run **c**) to 12 days (Run **d**), a dark time which is necessary to promote the third, final step when using the amine (Step 3 in Scheme 1). In this case, the secondary amines resulting from the first step can further react with unreacted diacrylate groups though a second Michael addition in order to increase the conversion of the diacrylate [11,18]. Irradiation of the composite was conducted for 2.5 s using an LED (Firejet FJ200, Phoseon, Hillsboro, USA) emitting at 395 nm with an irradiance of 2 W/cm^2^.

Dynamical mechanical analysis was made using the single-cantilever (16.47 mm) mode between −40 °C to 200 °C using a Q800 from TA Instruments (New Castle, DE, USA) on 30 × 11 mm^2^ samples at a rate of 2 °C/min, a deformation of 20 µm and a frequency of 1 Hz.

Cyclic thermomechanical tensile tests (CTTs) were measured as follows: The sample was first heated to the deformation temperature of *T_d_* = 110 °C and maintained at this temperature for 5 min. Then, the sample was stretched to the maximal strain of *ε_m_* = 0.5% at a strain rate of 0.5%/min. Under this imposed strain, the sample was cooled to the set temperature of *T_c_* = 20 °C and equilibrated for 5 min. The stress, *σ*, was then released by setting it to 0 Pa. Under no stress at *T_c_* the spontaneous recovery of the permanent shape can be recorded through the deformation, the result being defined as ε_u_. Finally, the sample is heated to *T_d_* under no stress (*σ* = 0 Pa) and the remaining deformation, *ε_p_*, after this recovery step is recorded. This thermomechanical cycle was repeated *N* times (1 < *N* < 4) for each sample.

## 3. Results and Discussion

**Figure 1:**
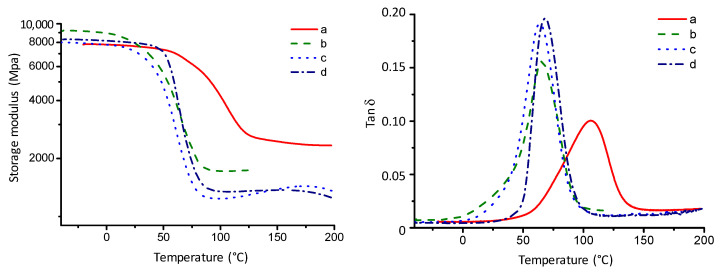
Changes in the storage modulus (**left**) and tan δ (**right**) monitored for Run **a**, Run **b**, Run **c** and Run **d**.

The fiber-reinforced polymers containing woven fibers and based on a combination of a Michael addition and free radical photopolymerization were first analyzed by DMA (Figure 1). All composites exhibited similar storage modulus values at the glassy plateau (E_g_) of about 7.8–8.2 GPa, except Run **b** which has a value of 9.1 GPa. At the rubbery state, Run **a** has a slightly higher storage modulus (E_r_) value (2.3 GPa) compared to Run **b** and Run **d** (1.2 and 1.7 GPa, respectively). The most interesting information can be obtained from tan δ curves. As can be seen in Table 1, the photopolymerization of the diacrylate alone (Run **a**) leads to a high T_max_^tanδ^ of 106 °C and a relatively large transition, with an FWHM of 46 °C.

When a Michael addition is added to the process as a preliminary step (Run **b** to Run **d**), it can be seen that the value T_max_^tanδ^ of the material decreases. This could be attributed to the presence of relatively long chains of the prepolymer created during the Michael addition reaction.

Table 1 shows that, in this case, the T_max_^tanδ^ decreases by about 40 °C. Interestingly, the values of the FWHM of the tan δ curve are reduced to about 14–21 °C for Run **b** to Run **d**. Comparing Run **b** and Run **c** shows that, at this stage, both thiol–Michael and aza–Michael addition reactions have the same effect: in these cases, the samples exhibit a more homogeneous polymer network than Run **a**. In addition, leaving the composite in the dark for 12 days (Run **d**) decreases the value of the FWHM of the tan δ curve. This feature is expected to yield good properties in terms of the SME.

**Figure 2:**
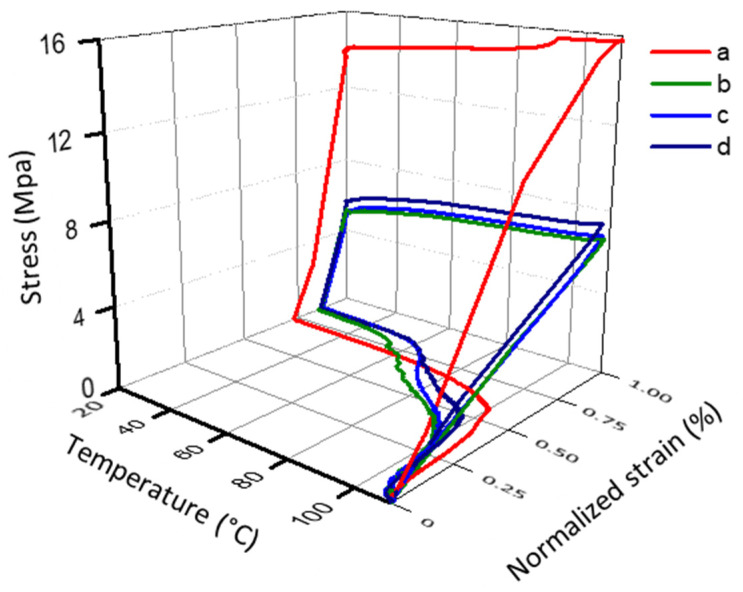
Resulting *σ*-*T*-*ε* curves obtained from cyclic thermomechanical tests of photocomposites Run **a**, Run **b**, Run **c** and Run **d**.

The shape memory effect of these composites was studied by cyclic thermomechanical single-cantilever tests using a strain-controlled procedure. CTTs monitoring the temperature, stress and strain of the composites are shown in Figure 2. Composites made with only EBAD, which is not an SMP, was used as a reference. Composites made with DA-EBAD have been analyzed one day after fabrication as well as twelve days later to study the impact of the third step on the SME.

Based on these results, shape fixity (*R_f_*) and shape recovery (*R_r_*) can be calculated using Equations (1) and (2), respectively:

(1) $$R_{f}\left(N\right)=\frac{\varepsilon_{u}\left(N\right)}{\varepsilon_{m}}$$

(2) $$R_{r}\left(N\right)=\frac{\varepsilon_{m}-\varepsilon_{p}\left(N\right)\;}{\varepsilon_{m}-\varepsilon_{p}\left(N-1\right)}$$

The results are reported in Table 2. The *R_r_* values during the first cycle show a good ability for all systems to recover their permanent shapes, at about 96%. Moreover, the values of cycles two to four are higher than 99% which show that the deformation/recovery cycle can be repeated without material fatigue. Nevertheless, the SME cannot be quantified only with the shape recovery. Indeed, it is important to confirm that the composite will not recover its permanent shape at Tc. Therefore, the shape fixity rate has an equal importance compared to the shape recovery rate. CTTs thus show that *R_f_* is equal to 73% for the sample made with free radical photopolymerization (Run **a**) only, while it is located between 87% and 90% for samples made with the thiol– or aza–Michael addition followed by FRP. The difference with Run **a,** made only by photopolymerization, can be explained as follows: During deformation, polymer chains adopt a new orientation to suit the stress. The presence of linear chains generated by the Michael addition will then allow this new orientation to be kept after the gelling of the polymer [21].

Fixity rates appear to be slightly lower for SMCs made with EBAD-DT and EBAD-DA systems compared to values obtained for SMPs, which were closed to 95%. Nevertheless, with rates located between 87% and 90% these SMCs presented satisfying results at the same level as those of thermal SMPs found in the literature [1]. Therefore, these systems demonstrated that it was possible to acquire SMCs using woven fibers thanks to a two-step system which involves photopolymerization.

Unlike the previous study led on SMPs [11], no significant differences can be seen between samples made with the DA-EBAD system analyzed one day or twelve days after their creation. This can be explained by the exotherm generated by the photopolymerization of the composite which allows for an increase in the conversion of acrylates. Therefore, there are not enough acrylate groups remaining to react with secondary amines and have an impact on the SME.

## 4. Conclusions

It was found that a combination of a Michael addition and free radical photopolymerization can be used to create woven fiber composites that exhibit the shape memory effect. The use of a dithiol or a diamine as a Michael reagent lead to an addition to the double bonds of acrylate, thereby creating a prepolymer. Thus, the photopolymerization of acrylate functions was conducted under an LED at 395 nm. The final composites are shown to exhibit a convincing shape memory effect, opening the way to create shape memory composites through a photopolymerization process.

## Figures and Tables

**Table 1:** Storage moduli measured at the glassy plateau (E_g_) and rubbery plateau (E_r_), maximum temperature T_max_^tan^^δ^ and the full width at half maximum (FWHM) of the tan δ curve.

	Run a	Run b	Run c	Run d
E_g_ (GPa)	7.8	9.1	7.8	8.2
E_r_ (GPa)	2.3	1.7	1.2	1.7
T_max_^tanδ^ (°C)	106	65	64	69
FWHM^tanδ^ (°C)	46	32	30	25

**Table 2:** Results of the thermocycle cantilever tests.

	Sample	*N* = 1		*N* = 2–4	
Run	Composition	$R_{f}\left(N\right)(\%)$	$R_{r}\left(N\right)(\%)$	$R_{f}\left(N\right)(\%)$	$R_{r}\left(N\right)(\%)$
**a**	EBAD	73.3	96.5	74.1	99.6
**b**	DT-EBAD	89.5	96.8	89.4	99.8
**c**	DA-EBAD t = 1 d	88.1	96.3	88.2	99.2
**d**	DA-EBAD t = 12 d	86.9	96.4	86.9	99.5

## Data Availability

The data presented in this study are available on request from the corresponding author. The data are not publicly available due to ongoing proprietary work but are available from the corresponding author on reasonable request.
